# Early Neurological Outcome of Young Infants Exposed to Selective Serotonin Reuptake Inhibitors during Pregnancy: Results from the Observational SMOK Study

**DOI:** 10.1371/journal.pone.0064654

**Published:** 2013-05-28

**Authors:** Nathalie K. S. de Vries, Christine N. van der Veere, Sijmen A. Reijneveld, Arend F. Bos

**Affiliations:** 1 Department of Paediatrics, Medical Centre Leeuwarden, Leeuwarden, The Netherlands; 2 Department of Paediatrics, Wilhelmina Hospital, Assen, The Netherlands; 3 Department of Health Sciences, University Medical Center Groningen, University of Groningen, Groningen, The Netherlands; 4 Division of Neonatology, Beatrix Children’s Hospital, University Medical Center Groningen, University of Groningen, Groningen, The Netherlands; The University of Queensland, Australia

## Abstract

**Background:**

Use of selective serotonin reuptake inhibitors (SSRI) during pregnancy is common while the effect on the infant’s neurological outcome is unknown. Our objective was to determine the effects of prenatal SSRI-exposure on the infants’ neurological functioning, adjusted for maternal mental health.

**Methods:**

A prospective observational study from May 2007 to April 2010. The study groups comprised 63 SSRI-exposed infants (SSRI group) and 44 non-exposed infants (non-SSRI group). Maternal depression and anxiety were measured using questionnaires. The main outcome measures during the first week after birth and at three to four months were the quality of the infants’ general movements (GMs) according to Prechtl and a detailed motor optimality score. We calculated odds ratios (ORs) and 95% confidence intervals (CIs) for abnormal GM quality in the SSRI and non-SSRI groups, and adjusted for maternal depression, anxiety, and other confounders. The study was registered under 53506435 in the ISRCTN.

**Findings:**

All infants were born around term. During the first week, abnormal GMs occurred more frequently in the SSRI group than in the non-SSRI group (59% versus 33%) and the median MOS was lower (13 versus 18). The OR for abnormal GMs in the SSRI versus the non-SSRI group was 3·0 (95% CI, 1.3 to 6.9) and increased after adjustment for confounders. At three to four months, more SSRI-exposed infants had monotonous movements (48% versus 20%) with lower median MOSs (26 versus 28). The OR for monotonous movements was 3·5 (95% CI, 1.5 to 8.6) and increased after adjusting for confounders.

**Interpretation:**

Prenatal exposure to SSRI had an adverse effect on early neurological functioning as reflected by GM quality, irrespective of maternal depression and anxiety, and other confounders. Physicians should take this into account in consultation with parents.

## Introduction

Women’s mental health problems during pregnancy are associated with adverse consequences for their infants, such as preterm birth, low birth weight, and may have long-term behavioural and developmental problems.[1]–[3] Since approximately 10% to 25% of pregnant women fulfil the diagnostic criteria for depression[1], [4], [5] and 7% to 18% those for anxiety disorder[6], [7], neonatal consequences of maternal mental health problems may have major effects on child health and development. Improved understanding of the influence of maternal depression on adverse outcome of pregnancies has resulted in prescription rates of antidepressant medication during pregnancy ranging from 2.0% in the Netherlands to 13.4% in the USA.[8], [9] Of all the antidepressants, selective serotonin reuptake inhibitors (SSRIs) are used most frequently.[8], [9] Since SSRIs readily cross the placenta [10] concern has risen about the short-term and long-term effects of prenatal exposure to SSRIs on the developing foetus.[4], [11]–[16].

We initiated an extensive prospective study, the Dutch SMOK trial (SSRIs in pregnant mothers, outcome of the kids) on the effects of exposure to SSRIs during pregnancy on the motor and cognitive outcome of children up to seven years of age. In the part of the study presented here, our aim was to determine the effects of prenatal SSRI exposure on early neurological functioning of the infant, adjusted for maternal mental health.

## Methods

### Study Design and Setting

We performed a prospective observational study on 107 mother-infant pairs from the catchment areas of two level-two hospitals in the northern part of the Netherlands (Medical Centre Leeuwarden and Wilhelmina Hospital Assen) and the nearby midwifery practices. This rural study area has a birth rate of about 2500 births per year. The study period was from May 2007 to April 2010.

Although our study was observational, it is registered with the Netherlands National Trial Registry under number 740, and its international standard randomised controlled trial number (ISRCTN) is 53506435.

### Ethics Statement

All parents (both mothers and fathers) gave their written informed consent on behalf of their infants. The Medical Ethics Committee of University Medical Center Groningen, the Netherlands, approved the study protocol and the consent procedure.

### Participants

**Figure 1:**
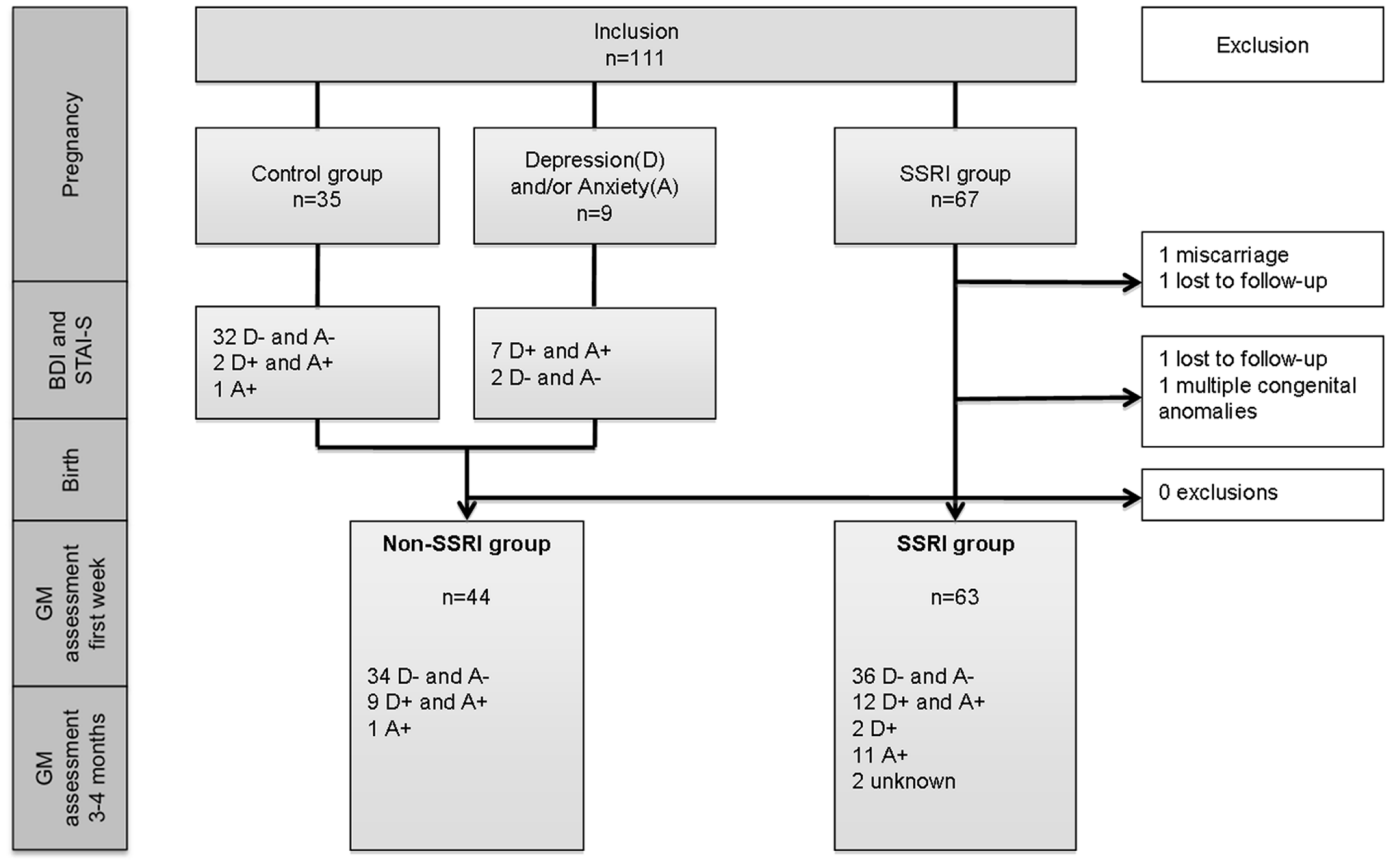
Flow sheet of inclusion and exclusion. A+ = anxiety. A− = no anxiety. BDI = Beck Depression Inventory. D+ = depression. D− = no depression. GM = general movement. STAI-S = State Trait Anxiety Inventory - State.

Initially the study comprised three study groups (Figure 1): 1. SSRI group. 2. Depression and/or anxiety group unmedicated. 3. Controls. During the inclusion process it appeared that the number of pregnant women who were referred to the second study group was low (two out of the nine women who said to have been diagnosed with depression and/or anxiety did not have a high score for depression or anxiety on the assessment questionnaires).The healthy control group appeared to also comprise women who had a positive score on depression or anxiety. We therefore merged the second and third groups into one non-SSRI group that consisted of pregnant women who did not use a SSRI. Inclusion was open. Women, in any stage of pregnancy and n both groups, were either self-referred, after reading about our study in the local newspapers, or referred by their gynaecologist, psychiatrist, midwife, or general practitioner).

The inclusion criteria for the SSRI group were SSRI treatment for depression and/or anxiety disorder during pregnancy and already taking medication before conception. Venlafaxine was considered to work as an SSRI if being dosed low [17], [18]; women using venlafaxine <200 mg were included in the SSRI group. Women, who had stopped taking medication before labour, remained in the SSRI group because they had taken SSRIs during the first trimester. The exclusion criteria were the use of psychotropic drugs other than SSRIs, anti-epileptic drugs, and multiple congenital anomalies of the infant. The inclusion criteria for the non-SSRI group were no psychotropic medication during pregnancy whatsoever. Exclusion criteria were the use of anti-epileptic drugs and multiple congenital anomalies of the infant.

Out of the 111 women initially included, one woman had a miscarriage, two women were lost to follow-up, and one child had multiple congenital anomalies (Figure 1). This resulted in 107 mother-infant pairs studied prospectively: 63 infants in the SSRI group and 44 infants in the non-SSRI group.

We had estimated that we needed to include at least 105 infants to be able to study the effects of SSRIs with two reference groups (i.e. two dummy variables) and five potential confounding variables on our primary outcome measure. This would yield seven dichotomous variables with 15 participants per comparison. This was a conservative estimate of ten events per dichotomized predictive variable plus a 50% buffer. [19].

### Measures and Procedures

#### SSRI medication

To be able to compare the doses of the different types of SSRIs that were used, we expressed these in dose equivalents as multiples of the standard Defined Daily Dose (DDD). [20] One DDD of the SSRIs used in this study was 20 mg for paroxetine, fluoxetine, and citalopram; 50 mg for sertraline; and 100 mg for venlafaxine. The dose equivalent was set to 1 to indicate standard exposure. For example, the dose equivalent was 2 for a woman using 40 mg of paroxetine per day.

#### Assessment of neurological functioning

We used the method of assessing neurological functioning by assessing the quality of an infant’s general movements (GMs) as developed by Prechtl [21] (see File S1). Two digital video recordings were made of each infant between days two and seven after birth, and again at three to four months. The recordings were made at the outpatient clinic or at home by either author CV or our research assistant. The infants lay partly dressed and supine. A recording of at least 5 minutes was made while the infant was in Prechtl’s state 4 (quiet wakefulness). [22] NV analysed the GMs offline according to Prechtl’s method. Although interrater reliability of GM assessment is high (89% to 93%) [21], in case of doubt, author AB was consulted to make the definitive judgment. NV and AB were blind as to the infants’ exposure status to SSRI.

#### Primary outcome measures: GMs and motor optimality score during the first week and at three to four months after term

The primary outcome measures were GM quality and a detailed motor optimality score (MOS) according to Prechtl’s optimality concept.[21], [23]–[25] The overall quality of the GM of the first recording was scored as either “normal” or “abnormal”.[21], [24] In case it was of abnormal we also scored whether the GMs were “poor repertoire” (PR), “chaotic” (Ch), “chaotic features” (ChF = infants with poor repertoire GMs with chaotic episodes), or “cramped synchronized” (CS). [24] Next, we assessed the MOS, which is based on a detailed analysis of the quality of the GMs on a continuous scale. The domains of the MOS at this age are overall quality (normal, PR, Ch, or CS), sequence, amplitude, speed, space, rotational components, onset/offset, and tremulous movements.[21], [24] The highest and most optimal score is 18, the lowest score is 8.

Overall GM quality of the second recording at three to four months was assessed as normal (which is the norm) or abnormal fidgety movements (FMs). Next, we assessed the concurrent motor repertoire in order to obtain a MOS. The domains of the MOS at this age are age-adequacy of the concurrent motor repertoire, presence and normality of individual movement patterns, presence and normality of individual postural patterns, and the nature of the movements of the concurrent repertoire (monotonous or not). The highest and most optimal score is 28, the lowest score is 5.[21], [26] Reduced complexity of the concurrent motor repertoire, resulting in monotonous movement, is related to minor neurological dysfunction in preterm infants. [23] We chose, therefore, to allocate a separate score for “monotonous movements”.

#### Other determinants: depression and anxiety

We assessed maternal depression and anxiety during the third trimester of pregnancy using the Beck Depression Inventory (BDI) [27] and the State Trait Anxiety Inventory (STAI) [7] for adults. At the infants’ age of three to four months, we re-assessed maternal depression and anxiety along with paternal depression and anxiety. The BDI is a self-reported questionnaire of 21 items measuring depressive symptoms (scores 0 to 63). Depression is defined by a BDI score of ≥13. [27] The STAI is a self-reported, 20-item questionnaire measuring temporary anxiety state conditions (STAI-S) and anxiety trait conditions (STAI-T) (scores 20 to 80). We chose to only use the STAI-S, assuming an anxiety state to be a possible reason for using SSRIs. Anxiety state, hereinafter referred to as “anxiety”, was defined as a STAI-S score of >40. [7].

### Statistical Analysis

Data were analysed using SPSS 19. We first assessed the background characteristics of the women in the SSRI and non-SSRI groups. Second, we assessed the association of SSRI exposure with the quality of the infants’ GMs during the first week (abnormal/normal) and at three to four months of age (monotonous movements/no monotonous movements). For the MOS we used the Mann-Whitney test. Subsequently, we assessed the association of SSRI exposure with abnormal GM quality during the infants’ first week and monotonous GMs at three to four months by calculating odds ratios (ORs).

We adjusted for possible confounders and calculated adjusted ORs (aORs) by using a multivariate logistic regression model. The confounders we considered were maternal depression and anxiety during the third trimester of pregnancy, low-level maternal education (lower than vocational education), low gestational age (below 38 weeks), low birth weight (below 2750 g), and sex. For the GMs at the age of three to four months we also considered paternal depression and anxiety as possible confounders. Because depression and anxiety overlap, we constructed two models: we entered only one of the entities regarding maternal mood in each model apart from the other confounders, giving aOR_depr+_ and aOR_anx+_. Differences were considered statistically significant at *p*<.05.

## Results

### Background Characteristics

In Table 1 we present the background characteristics and other determinants including those related to depression and/or anxiety. There was an overlap in the type of mood disorder. In the SSRI group only 3% of the women had depression, 22% had anxiety, and 20% had both. In the non-SSRI group this was 0%, 3%, and 21%, respectively.

**Table 1:** Background characteristics of women and infants.

	SSRI group (n = 63)	Non-SSRI group (n = 44)
**Women’s general characteristics**		
Age at labour, years, median (min-max)	31 (21–42)	32 (22–39)
Caesarean section, n (%)	6 (10)	4 (9)
Low-level education*, n (%)	26/57 (46)	17/43 (40)
Smoking, n (%)	11/58 (19)	4/43 (9)
Cigarettes per day among smokers, median (min-max)	6 (1–20)	5 (5–10)
Alcohol use >2 units/wk, n (%)	3/61 (5)	0/42 (0)
**Maternal psychopathology during pregnancy**		
BDI score, median (min-max)	7 (0–34)	4 (0–35)
Depression, n (%)	14/61 (23)	9 (21)
STAI-S score, median (min-max)	37 (20–76)	30 (20–72)
Anxiety state, n (%)	23/61 (38)	10 (23)
**Maternal psychopathology at infant’s age 3–4 months**		
BDI score, median (min-max)	6 (0–32)	3 (0–27)
Depression, n (%)	15/59 (25)	6 (14)
STAI-S score, median (min-max)	34 (21–68)	26 (20–75)
Anxiety state, n (%)	23/59 (39)	7 (16)
**Infants’ general characteristics**		
Male sex, n (%)	27 (43)	22 (50)
Gestational age, weeks, median (min-max)	39·1 (36·7–42·7)	40·0 (37·3–41·7)
Preterm, n (%)	2 (3)	0 (0)
Birth weight, grams, median (min-max)	3455 (2225–5500)	3830 (2500–4750)
Birth weight <p10, n (%)	10 (16)	3 (7)
Apgar score ≤5 at 5 min, n (%)	2 (3)	0 (0)
Breastfed, n (%)	25/59 (42)	–
Breastfed >50% in months, median (min-max)‡	4 (1–14)	–
**Paternal psychopathology at infant’s age 3 to 4 months**		
BDI score, median (min-max)	1 (0–19)	1 (0–10)
Depression, n (%)	4/52 (8)	0/42 (0)
STAI-S score, median (min-max)	26 (20–58)	29 (20–48)
Anxiety state, n (%)	8/50 (16)	6/41 (15)

− not applicable.

* low-level education = educational level lower than vocational education.

‡ The number of months the infant received more than 50% of the total amount of milk from breastmilk.

BDI = Beck Depression Inventory.

STAI-S = State Trait Anxiety Inventory-State.

The BDI score during pregnancy correlated highly with the BDI score at the infants’ age of three to four months (Spearman’s rho = 0.62; *p*<.001); the same was true for the STAI-S score (Spearman’s rho = 0.72; *p*<.001).

One of the two infants with a low Apgar score had a normal umbilical pH and fully recovered after several days. The other infant suffered from birth asphyxia with Apgar scores of 0 and 0 after 1 and 5 minutes. This infant received hypothermia treatment at a tertiary neonatal intensive care centre and was hospitalised for several weeks. Of the remaining infants the first three months were uneventful considering diseases that might influence neurological functioning like meningitis, sepsis, or neurotrauma.

### Characteristics of SSRI Medication

In Table 2 we present the data about the type of SSRI medication, daily doses and dose equivalents.

**Table 2:** Characteristics of SSRI medication.

Type of SSRI used	n (%)	Daily dose (range)	Dose equivalent* (range)	n (%) below/equal/above DDD**
Paroxetine	27 (43)	10–40	0.5–2	5 (19)/18 (67)/4 (15)
Cipramil	14 (22)	10–30	0.5–1.5	2 (14)/11 (79)/1 (7)
Venlafaxine	10 (16)	37.5–150	0.38–1.5	3 (30)/0 (0)/7 (70)
Fluoxetine	8 (13)	10–40	0.5–2	2 (25)/5 (63)/1 (13)
Sertraline	2 (3)	50–100	1–2	0 (0)/1 (50)/1 (50)
Changed medication	2 (3)			
Stopped medication	4 (6)			

* Dose equivalent = Daily dose divided by Defined Daily Dose.

** DDD = Defined Daily Dose: paroxetine = 20 mg; cipramil = 20 mg; venlafaxine = 100 mg; fluoxetine = 20 mg; sertraline = 50 mg.(20).

### Assessments of Neurological Outcomes

For 107 infants, 209 recordings were made out of the planned 214. In the SSRI group we missed making five recordings in the first week due to either the parents or the nurses forgetting to inform us of the infant’s birth. The median age (minimum-maximum) at first recording was the same for both groups, i.e. 3.0 days (2–7). The postnatal age in weeks after term at second recording for the SSRI group was 14.0 (12–20) and 14.0 (11–17) for the non-SSRI group.

### Neurological Outcome as Assessed by GMs in Relation to SSRI Exposure

#### GM quality during the first week

Table 3 shows that abnormal GMs during the first week were observed nearly twice as often in the SSRI as in the non-SSRI group. The crude OR for abnormal GMs in the SSRI versus the non-SSRI group was 3.0 (95%CI, 1.3–6.9, *p* = .008) (Table 4).

**Table 3:** Motor performance in the SSRI and non-SSRI group of infants.

		SSRI group (n = 63)	Non-SSRI group (n = 44)	Statistical significance and *p* value
**GMs during first week**				
**GM quality**		n = 58*	n = 44	
	Abnormal, n (%)	34 (59)	14 (33)	0.009
	Poor repertoire, n (%)	30 (52)	14 (33)	0.11
	Chaotic, n (%) / ChF, n (%)	4 (7) / 11 (19)	0 (0) / 1 (2)	0.14 / 0.012
	Cramped synchronized, n (%)	0 (0)	0 (0)	
**MOS**, median (min-max)		13 (9-18)	18 (10-18)	<0.001‡
	Monotonous sequence, n (%)	36 (62)	13 (30)	0.001
	Amplitude abnormalities, n (%)	13 (22)	3 (7)	0.052
	Speed abnormalities, n (%)	26 (45)	16 (36%)	0.042
	Not using up full space, n (%)	11 (19)	0 (0)	0.002
	No rotations, or jus a few, n (%)	28 (31)	7 (16)	0.001
	Abrupt onset and/or offset, n (%)	16 (28)	5 (11)	0.051
	Tremulous movements, n (%)	35 (60)	13 (30)	0.012
**GMs at 3 to 4 months of age**				
**GM quality**		n = 63	n = 44	
	Abnormal quality, n (%)	3 (5)	0 (0)	0.27‡
	Abnormal fidgety, n (%)	2 (3)	0 (0)	
	Absent fidgety, n (%)	1 (2)	0 (0)	
**MOS**, median (min-max)		26 (7–28)	28 (21–28)	0.035‡‡
	Monotonous movements, n (%)	30 (48)	9 (20)	0.005‡

ChF  =  Chaotic features.

GM  =  general movement.

MOS  =  motor optimality score.

* Five recordings were missing in the SSRI group.

‡ Fisher exact test.

‡‡ Mann-Whitney test.

**Table 4:** Crude and adjusted odds ratios for abnormal general movements in the SSRI versus non-SSRI group.

	Crude OR; 95% CI (*p* value)	aOR_depre+_; 95% CI (*p* value)	aOR_anx+_; 95% CI (*p* value)
Abnormal GMs during the first week	3.0; 1.3–6.9 (0.008)	4.1; 1.6–10.5 (0.003)	4.2; 1.6–10.9 (0.003)
Monotonous movements at 3 to 4 months of age	3.5; 1.5–8.6 (0.005)*	6.4; 2.1–19.2 (0.001)*	5.8; 1.9–17.7 (0.002)*

CI = confidence interval.

GA = gestational age.

GM = general movement.

OR = odds ratio.

aOR_depre+_ = adjusted odds ratio, corrected for maternal depression during 3^rd^ trimester, low-level maternal education (lower than vocational education), gestational age below 38 weeks, birth weight below 2750 g, and sex.

aOR_anx+_ = adjusted odds ratio, corrected for maternal anxiety during 3^rd^ trimester, low-level maternal education (lower than vocational education), gestational age below 38 weeks, birth weight below 2750 g, and sex.

* also corrected for paternal depression and anxiety at the infant’s age of 3 to 4 months.

ChF GMs were seen more often in the SSRI than in the non-SSRI group (Table 3). Chaotic GMs were rare and only seen in the SSRI group.

The MOSs were significantly lower in the SSRI group, and scores on all subdomains were more often abnormal. The range of amplitude abnormalities was either too small or too large. Similarly, speed abnormalities were also either too low or too high.

In the SSRI group, a lower MOS was associated with a higher dose equivalent (Spearman’s Rho −0.361, *p* = 0.005).

#### GM quality at three to four months

At the age of three to four months only three infants had abnormal FMs (Table 3). All three belonged to the SSRI group. MOSs were significantly lower in the SSRI group. The concurrent repertoire showed monotonous GMs significantly more often in the SSRI group. The crude OR for monotonous movements in the SSRI group versus the non-SSRI group was 3.5 (95%CI, 1.5–8.6, *p* = .005) (Table 4).

In the non-SSRI group MOS during the first week related to MOS at three to four months (Spearman’s rho 0.305, *p* = 0.044). This was not the case in the SSRI group.

In the SSRI group, the MOS was not related to the dose equivalent.

### Motor Performance in Relation to Confounders

**Figure 2:**
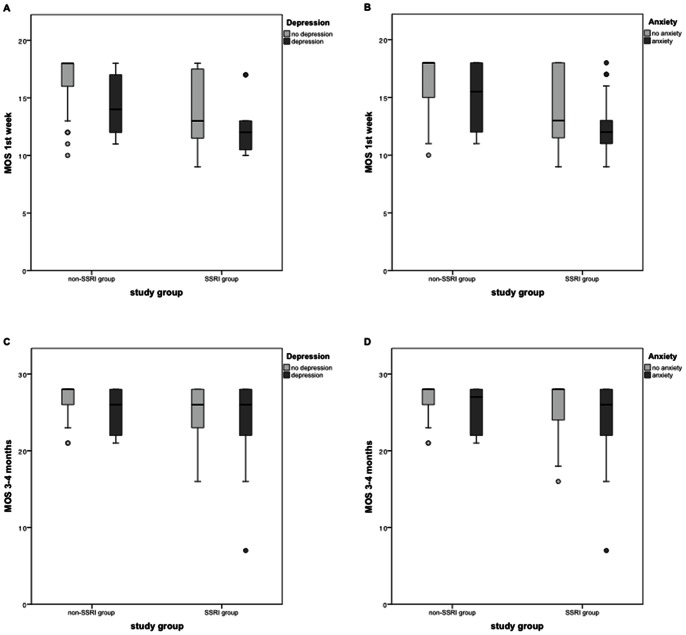
Motor optimality scores in the SSRI and non-SSRI groups, clustered by maternal depression and anxiety. Motor optimality scores during the first week (A, B) and at 3 to 4 months (C, D) in the SSRI and non-SSRI groups, clustered by maternal depression and anxiety during pregnancy. The data in the graphs are presented as box-and-whisker plots. The boxes represent the individual values between the 25^th^ and 75^th^ centile (interquartile range, IQR), the whiskers represent the range of the values, with the exception of outliers. Significant differences were tested with the Mann-Whitney test. MOS = motor optimality score.

Table 4 shows the ORs for abnormal GMs in the SSRI versus the non-SSRI group after adjusting for potential confounders. Figure 2 illustrates the median MOS during the first week (Figures 2A and 2B) and at three to four months (Figures 2C and 2D) in the SSRI and non-SSRI groups categorized according to maternal depression and anxiety.

## Discussion

We demonstrated that prenatal SSRI exposure has a negative effect on the quality of GMs and as such it is a reflection of the negative effect these antidepressants have on early neurological outcome. SSRI exposure was strongly associated with abnormal GMs and lower MOSs during the first week after birth, and a monotonous motor repertoire and lower MOSs at the age of three to four months. This association became even more pronounced after we had adjusted for the confounders maternal depression and anxiety, maternal education, the infants’ gestational age, birth weight and sex, and paternal depression and anxiety.

Neurological effects of prenatal SSRI exposure could be due to serotonin withdrawal, to direct serotonergic effects, or to teratogenic effects of the SSRI on the foetal brain. Since the motor repertoire during the first week was not associated with the motor repertoire at three to four months in the SSRI group, we speculated that the effects observed during the first week were possibly due to all of the above mentioned factors, while the effects seen at three to four months were probably due to permanent teratogenic effects of the SSRI on the developing brain.

In the first week, we observed a different spectrum of symptoms in SSRI-exposed infants than in the non-exposed group that was dose-related. This spectrum has been variously named SSRI-induced neonatal-behaviour-syndrome, poor neonatal adaptation, or prenatal antidepressant exposure syndrome.[10], [14], [15], [28]–[31] In SSRI-exposed foetuses, a dose-related response was also found by Mulder *et al*. [32] They reported that abnormal foetal neurobehavioral development throughout gestation was associated with standard and high doses of SSRIs. [32].

Of special interest to us was the dualism of disturbances in GMs between the infants, i.e. monotonous movements and few rotations on the one hand, and tremulous movements and chaotic features on the other hand. Moreover, both high and low amplitudes and speeds were seen in SSRI-exposed infants. Zeskind et al. described both lower arousal (under-stimulation) and higher arousal (hyperexcitability) in such neonates. [30] A similar dualism is described by Mosos-Kolko et al. in adults with SSRI-induced behavioural syndromes. [33] As we did in our study, Salisbury et al. also discriminated between the effects of SSRI exposure and maternal mood on neonatal neurobehaviour between days 1 to 21 after birth. [28] In the infants of women with major depressive disorder, who had received SSRI treatment during pregnancy, they found a lower quality of movement and more signs of central nervous system stress. In contrast, in infants of women with only major depressive disorder, without SSRIs they found the highest quality of movements but lower attention scores. Our findings confirm these different spectra at a more detailed level.

At the age of three to four months, 104 out of the 107 infants showed normal overall GM quality, i.e. normal FMs. Only three infants who had been exposed to SSRIs had abnormal FMs. We know from other GM studies that abnormal GM quality at this age is highly predictive of later neurological impairment. [21] We speculated that an overall assessment of GM quality at this age might not be sensitive enough to detect minor neurological sequelae. For this purpose, the MOS and the concurrent repertoire might be more revealing. When considering the concurrent repertoire, we found that monotonous movements were seen twice as often and that the MOS was significantly lower in the exposed infants compared to the non-exposed infants. Previously, it was reported that an abnormal concurrent repertoire is related to minor neurological dysfunction in preterm-born infants. [23] We speculated, therefore, that the lower MOSs and the monotonous movements in our SSRI-exposed infants could be indicative of permanently altered brain function due to exposure to these antidepressants. Studies on the early effects of SSRI exposure on infants aged three to four months are few. Only Oberlander et al. described altered neonatal acute pain response after SSRI exposure during pregnancy that persisted in two-month-old infants in a prospective study. [34].

We recognize several limitations of our study. First, open inclusion may have led to selection bias. The inclination to participate in a study considering the possible negative effects of SSRI exposure depends on the motivation of the parents. It is unlikely, however, that this affected our findings since the participants were unaware of the outcomes at the moment of inclusion. Second, since the control group was not a healthy control group, we cannot generalize the outcome of the infants in the non-SSRI group to the general population. Although the incidence of depression in the non-SSRI group was not extremely high (21%) compared to the literature (10% to 25%) [1], [4], [5], the incidence of anxiety was high (23%) compared to the literature (7% to19%). [6], [7] It was our aim to discriminate between effects of SSRI exposure and effects due to maternal depression or anxiety or both on the infants’ neurological functioning. A high incidence of depression and anxiety in the non-SSRI group increased our power to adjust for this confounder. A third limitation was that we assessed maternal depression and anxiety using validated screening questionnaires rather than a structured psychiatric interview, the golden rule for diagnosing depression and anxiety. Possibly, as a consequence, the incidence of depression and anxiety in our study groups was slightly overestimated, which may have diluted estimates somewhat. Finally, we assessed maternal mood during pregnancy in the third trimester only. Therefore, we might have missed depression and anxiety during the first or second trimester, while stress early in pregnancy can also affect neurological outcome of the infant.

Our results have important implications. Clinically, physicians should inform parents about the advantages and disadvantages of taking SSRIs during pregnancy, and about the fact that SSRIs have an independent effect on their infant’s developing brain. SSRI exposure can influence neurological function of the foetus, not transiently, as seen during the first days after birth, but also later, at least at the age of three to four months. Physicians should be aware that prescriptions should be limited to those women who have an indication for prescription after careful evaluation by professionals followed by pre-pregnancy advice in case of child wish. We plea for centres with outpatient facilities in which paediatricians, obstetricians and psychiatrists collaborate.

As far as research is concerned, our study needs to be replicated, preferably including women with anxiety and depression disorders confirmed by a structured psychiatric interview. In addition, data on long-term neurological outcome of the infants are needed. For this purpose we are planning to reassess the children at two-and-a-half and seven years of age as part of this on-going SMOK trial.

In conclusion, prenatal exposure to SSRIs probably has negative effects on the early neurological functioning of the infant, irrespective of maternal mental state. Given the high rates of SSRI prescriptions to pregnant women (2% to13%)[8], [9], this may have considerable consequences for public health.

## Supporting Information

File S1 **Assessment of neurological functioning according to Prechtl.**
(DOCX)

File S2 **STROBE checklist for cohort studies.**
(PDF)
