# Supplementary material for: Early Neurological Outcome of Young Infants Exposed to Selective Serotonin Reuptake Inhibitors during Pregnancy: Results from the Observational SMOK Study
Source: PLoS One. 2013 May 28;8(5):e64654. doi: 10.1371/journal.pone.0064654 (PMC3665535; doi:10.1371/journal.pone.0064654)
Supplement: File S1 — Assessment of neurological functioning according to Prechtl. (DOCX) [file pone.0064654.s001.docx]

**Assessment of neurological functioning according to Prechtl.**

We used Prechtl’s method for assessing the quality of general movements (GMs) to assess neurological functioning.(21) GMs are complex, endogenously generated movements, i.e. movements not triggered by sensory input, and they involve the whole body. They are present from early foetal life until the end of the second month after term. By and large, GMs look similar from early foetal life until the end of the second month after term, although minor age-related differences do exist. At term age, GMs have a writhing character. Typically, they are ellipsoid and create the impression that the infant is writhing. In case the nervous system is impaired, GMs lose their complexity and the infant’s movement repertoire becomes poor. At around two months after term the writhing GMs gradually disappear and fidgety movements (FMs) emerge. These movements are present up to five to six months after term. FMs are characterized by small amplitude, moderate speed, and variable acceleration of neck, trunk, and limbs in all directions, and are continuously present in the awake infant. Abnormal FMs at this age are highly predictive of later neurological impairments. The absence of FMs, in particular, is closely associated with cerebral palsy (CP).(21) The presence of FMs indicates a good neurological prognosis. FMs coexist with other movements, the concurrent motor repertoire, including manipulating clothing, reaching, leg lifting, head rotation, and postural control. Reduced complexity of the concurrent repertoire is related to minor neurological dysfunction in preterm infants.(23,26)
